# Trastuzumab Potentiates Antitumor Activity of Thiopyrano[2,3-*d*]Thiazole Derivative in AGS Gastric Cancer Cells

**DOI:** 10.3390/molecules29215117

**Published:** 2024-10-30

**Authors:** Piotr Roszczenko, Olga Klaudia Szewczyk-Roszczenko, Agnieszka Gornowicz, Robert Czarnomysy, Andrii Lozynskyi, Krzysztof Bielawski, Roman Lesyk, Anna Bielawska

**Affiliations:** 1 Department of Biotechnology, Medical University of Białystok, Kilińskiego 1, 15-089 Białystok, Poland; 2 Department of Synthesis and Technology of Drugs, Medical University of Białystok, Kilińskiego 1, 15-089 Białystok, Poland; 3 Department of Pharmaceutical, Organic and Bioorganic Chemistry, Danylo Halytsky Lviv National Medical University, Pekarska 69, 79010 Lviv, Ukraine

**Keywords:** anticancer, gastric cancer, 4-thiazolidinone, anti-HER2, trastuzumab, combined therapy

## Abstract

Gastric cancer remains a significant therapeutic challenge, highlighting the need for new strategies to improve treatment efficacy. This study investigates the potential of combined therapy with the novel Thiopyrano[2,3-*d*]Thiazole derivative LES-6400 and the anti-HER2 antibody trastuzumab in AGS gastric cancer cells. The antitumor effects of the combined therapy were evaluated using various techniques, including the MTT assay for cell viability, [^3^H]-thymidine incorporation for DNA synthesis, and flow cytometry to assess apoptosis (Annexin V-FITC/PI staining), mitochondrial membrane potential (MMP), and inflammatory cytokine levels. ELISA was employed to measure the levels of IL-6, p53, and cytochrome C. The combination of LES-6400 (1 µM) and trastuzumab (10 µg/mL) demonstrated superior antitumor activity compared to monotherapy with either agent in AGS gastric cancer cells. The combination therapy enhanced apoptosis, presumably by inducing oxidative stress in the cells and disrupting mitochondrial membrane potential. Additionally, a significant increase in p53 protein levels and modulation of interleukin levels, including a marked reduction in IL-6 levels, were observed, suggesting an impact on apoptotic and inflammatory responses. These findings indicate that the combined use of LES-6400 and trastuzumab is a promising therapeutic strategy for gastric cancer, warranting further investigation into the mechanisms of action and potential clinical applications of this combined approach.

## 1. Introduction

Despite advances in science and medicine, cancer continues to pose a serious threat to human life. In 2022 alone, cancer was the direct cause of 9,743,832 deaths, with 2,296,840 attributed to breast cancer and 968,784 to stomach cancer [1,2,3]. Because of these alarming statistics, researchers worldwide are intensively searching for new drugs and strategies to treat cancer. Current treatments are often not effective enough; a narrow therapeutic index and poor selectivity are the main causes of their side effects [4]. Continuing advances in research in medicinal chemistry, molecular biology, and cancer genetics offer hope for more effective treatments for this “epidemic” of the modern world.

**Figure 1:**
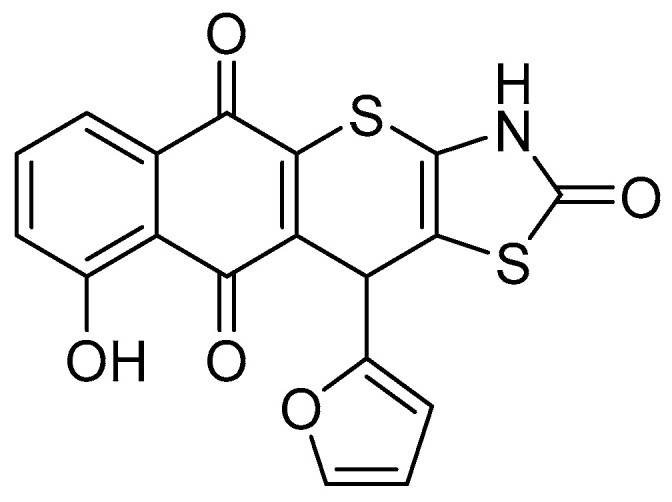
Structure of studied juglone-based Thiopyrano[2,3-*d*]Thiazole derivative LES-6400 (11-(furan-2-yl)-9-hydroxy-3,11-dihydro-2*H*-benzo[6,7]thiochromeno [2,3-*d*]thiazole-2,5,10-trione).

Heterocyclic compounds provide a solid foundation for anticancer drug discovery [5,6,7]. To date, a wide range of biological activities of thiazole derivatives, their structural analogues, as well as their condensed derivatives, have been investigated. Among these compounds, thiopyranothiazole derivatives, which combine thiazole and thiopyran fragments in one structure, are of special interest [8]. Molecules of this class possess a wide spectrum of biological activities, such as antiviral [9], antitrypanosomal [10], antimicrobial [11], and anticancer [12]. For some Thiopyrano[2,3-*d*]Thiazole derivatives, several mechanisms have been identified that ensure the realization of their biological activities, particularly the inhibition of transforming growth factor beta (TGF-β) [13], tubulin polymerization [14], and activation of PPARγ receptors [15]. The use of various synthetic methodologies, such as *hetero*-Diels-Alder reactions, Michael addition, and heterocyclizations based on Knoevenagel condensation, facilitates the synthesis of a wide range of functionally substituted thiopyranothiazole derivatives. These approaches also allow for the incorporation of natural compound fragments, such as citral, cinnamic acids, and juglone, into the Thiopyrano[2,3-*d*]Thiazole moiety. Juglone (5-hydroxy-1,4-napthoquinone) is a compound of natural origin found mainly in parts of plants of the Juglandaceae family. Juglone itself has properties including anticancer, antioxidant, antimicrobial, and vertebrate sedative [15,16]. The synthesis of Thiopyrano[2,3-*d*]Thiazoles with juglone fragment in molecules yielded derivatives with more potent anticancer activity than their synthetic precursors. Additionally, the juglone-based Thiopyrano[2,3-*d*]Thiazole derivative LES-6400 (Figure 1) demonstrated the strongest anticancer cytotoxicity within the compound series, comparable to doxorubicin. After 72 h of incubation, in MTT assay, the IC_50_ values for various cancer cell lines were as follows: 0.6 ± 0.24 μM for colon cancer (HCT-116), 2.37 ± 0.43 μM for HCT-116 p53 (−/−), 3.08 ± 0.41 μM for breast cancer (MCF-7), 5.98 ± 0.41 μM for leukemia (K562), and 0.75 ± 0.29 μM for cervical cancer (KB3-1). In contrast, for normal cells, the IC_50_ values were 37.16 ± 0.56 μM for epidermal keratinocytes (HaCaT), 2.59 ± 0.13 μM for mouse macrophages (J774.2), and 6.07 ± 0.41 μM for pseudonormal mouse fibroblasts (NIH 3T3). The IC_50_ values of LES-6400 against normal and pseudonormal cell lines were notably higher than those for doxorubicin, amounting to 4.58 ± 0.78 μM, 0.56 ± 0.98 μM, and 0.72 ± 1.00 μM, respectively. This derivative was found to activate apoptosis—programmed cell death—via both intracellular and extracellular pathways. It induced cell cycle arrest at the G2/M phase and inhibited DNA and RNA synthesis in cancer cells. Furthermore, the derivative interacted with DNA through electrostatic interactions and intercalation. Importantly, LES-6400 showed low acute toxicity in C57BL/6 mice [13].

HER2 (human epidermal growth factor receptor 2) is one of the four cell membrane receptor tyrosine kinases (RTKs) whose gene is located on chromosome 17q12.1. HER2 is a transmembrane glycoprotein consisting of an extracellular ligand-binding domain, a transmembrane domain, and an intracellular tyrosine kinase catalytic domain [17]. HER2 overexpression contributes to tumorigenesis by forming homodimers or heterodimers with other members of the RTK family, activating signaling pathways such as PI3K/Akt/mTOR and MAPK, and promoting cell proliferation, survival, and angiogenesis. HER2 overexpression in HER-2 positive breast cancer is associated with a very poor prognosis compared to HER-2 negative breast cancer [18]. In particular, in HER-2-positive breast cancer, it is associated with a worse prognosis compared to HER-2-negative cases, meaning a higher risk of local tumor growth and metastasis [19]. Trastuzumab (Herceptin^®^) is a humanized IgG1 monoclonal antibody approved for the treatment of early HER-2 positive breast cancer, as adjuvant and neoadjuvant therapy, and for the treatment of metastatic HER-2 positive gastric adenocarcinoma of esophagogastric junction disease [20]. Trastuzumab, both as monotherapy and in combination with chemotherapy, has shown benefit in patients with HER-2-positive metastatic breast cancer, prolonging time to disease progression and overall survival. Phase III studies have confirmed that the addition of trastuzumab to standard adjuvant chemotherapy significantly reduces the risk of disease recurrence or death [21]. Clinical benefit has also been demonstrated in other HER-2-overexpressing cancers, most notably gastric cancer, where trastuzumab prolonged survival compared with the control arm. These results led to the approval of trastuzumab as a first-line treatment for gastric cancer with HER-2 [22].

The principal role of HER2 is to suppress apoptosis to promote cell survival, leading to uncontrolled proliferation and tumor growth [23]. The intrinsic apoptotic pathway is initiated by intracellular signals that converge at the mitochondrial level in response to various stress factors, including ionizing radiation and chemotherapeutics. Intrinsic stimuli, such as DNA damage or increased levels of reactive oxygen species (ROS), result in the activation of this pathway. This results in the activation of proapoptotic proteins belonging to the Bcl-2 family, such as Bax and Bak, and the inactivation of anti-apoptotic proteins, including Bcl-2, Bcl-xL, and Mcl-1. Subsequently, this results in a disruption of the mitochondrial membrane potential, which in turn leads to the release of apoptogenic proteins into the cytosol. Among these is cytochrome C, which plays a pivotal role in the activation of mitochondrial-dependent apoptosis. Cytochrome C binds to cytosolic Apaf-1 (apoptosis protease-activating factor-1) to form the apoptosome, which activates a cascade of procaspase-9, followed by executioner caspases-3, -6, and -7, which are responsible for cleaving cellular components and apoptosis [24,25].

Given the extensive study of the juglone-based Thiopyrano[2,3-*d*]Thiazole derivative LES-6400 (Figure 1), which has demonstrated the most promising cytotoxic activity among the series of the synthesized compounds displayed relatively low toxicity towards normal and pseudonormal cell lines, as well as low acute toxicity in vivo [13]. In this study, we have chosen to evaluate their activity against several previously untested gastric, colon, and breast cancer cell lines.

Anti-HER-2 antibodies are used in combination therapy for the treatment of breast and gastric cancer [22]. We decided to evaluate the advantages of combination therapy with the LES-6400 derivative and antibody anti-HER-2 compared to monotherapies with the drug alone and with the antibody alone.

## 2. Results

### 2.1. LES-6400 Is Highly Cytotoxic to Cancer Cell Lines

Based on preliminary studies previously published by our team, the LES-6400 derivative has demonstrated cytotoxic activity [13]. We decided to focus our subsequent research on gastrointestinal cancers (AGS, DLD-1, HT-29) and breast cancers (MDA-MB-231, MCF-7, HCC1954). After 24 h of exposure to the tested compound in the MTT assay, we obtained IC_50_ values ranging from 1.88 to 10.02 μM (Table 1). For further studies, we selected the AGS and HCC1954 cell lines, as the compound exhibited the highest activity against them.

### 2.2. The Combination Treatment with the LES-6400 and the Trastuzumab Shows a More Potent Cytotoxic Effect on Gastric Cancer Cells

**Figure 2:**
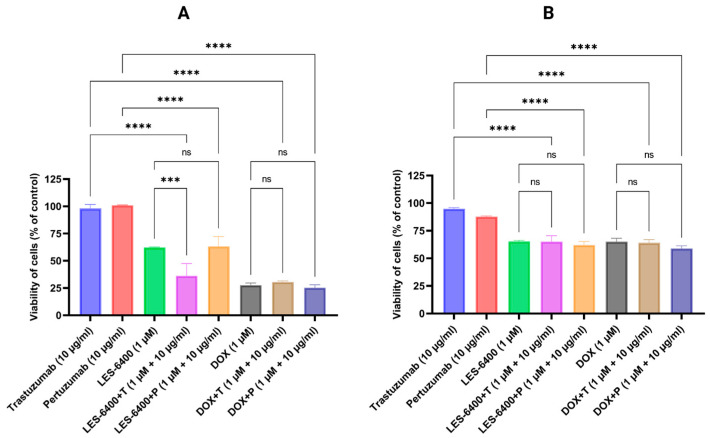
Cell survival of AGS gastric cancer cells (**A**) and HCC1954 breast cancer cells (**B**) was assessed after 24 h of treatment with LES-6400 alone, trastuzumab alone, pertuzumab alone, doxorubicin alone (DOX), and the following combinations: LES-6400 + trastuzumab (LES-6400 + T), LES-6400 + pertuzumab (LES-6400 + P), DOX + trastuzumab (DOX + T), and DOX + pertuzumab (DOX + P). Means ± SD, are shown with *N* = 3. ANOVA tests were used to demonstrate differences between cells treated with compounds alone and the combination of compounds and antibodies. **** *p* ≤ 0.0001; *** *p* ≤ 0.001; ns *p* > 0.05—not statistically significant.

After selecting the cell lines for further study, we evaluated the effect of combination treatment with the test compound and anti-HER2 antibodies. The combination treatment proved to be more effective in the AGS gastric cancer cell line. AGS cell survival relative to the control was 36.17% ± 11.27% for the combination of LES-6400 (1 μM) and trastuzumab (10 μg/mL), compared to 62.19% ± 0.44% for LES-6400 (1 μM) alone and 97.95% ± 3.79% for trastuzumab (10 μg/mL) alone. We observed no change in cell survival when doxorubicin was combined with the antibodies. Additionally, we found that the combination LES-6400 (1 μM) and pertuzumab (10 μg/mL) did not enhance the antitumor effect (Figure 2).

**Figure 3:**
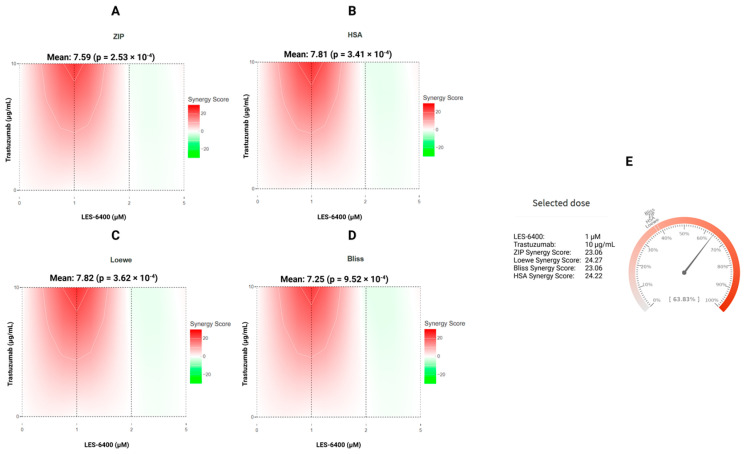
The synergy distribution between the juglone-based Thiopyrano[2,3-*d*]Thiazole LES-6400 and the anti-HER2 trastuzumab antibody against the AGS gastric cancer cell line is demonstrated for ZIP (**A**), HSA (**B**), Loewe (**C**), and Bliss (**D**). Additionally, the presented Synergy Barometer for LES-6400 + T (1 μM + 10 μg/mL) is illustrated (**E**).

To corroborate the findings, the synergistic effect of LES-6400 and trastuzumab against the AGS gastric cancer cell line was calculated using the online tool SynergyFinder+. The mean synergy scores for LES-6400 + T were 7.81 (HSA), 7.82 (Loewe), 7.25 (Bliss), and 7.59 (ZIP), respectively (Figure 3A–D). The *p* values were all less than 0.05, indicating that the observed effects were statistically significant and that the combination exhibited additive or mildly synergistic properties. Moreover, focusing on the combination of LES-6400 at a concentration of 1 μM and trastuzumab at a concentration of 10 μg/mL, a combination index (CI) of 0.77 was calculated. The results indicated that the combination of LES-6400 at a concentration of 1 μM and trastuzumab at a concentration of 10 μg/mL exhibited very strong synergistic properties, as evidenced by the following values: 23.06 for the ZIP, 24.27 for the Loewe, 23.06 for the Bliss, and 24.22 for the HSA, as calculated by the ‘Synergy Barometer’ (Figure 3E) [26]. Given these results, we decided to continue further studies in the AGS gastric cancer cell line.

### 2.3. Trastuzumab Enhances the Anti-Proliferative Effect of the Compound LES-6400

**Figure 4:**
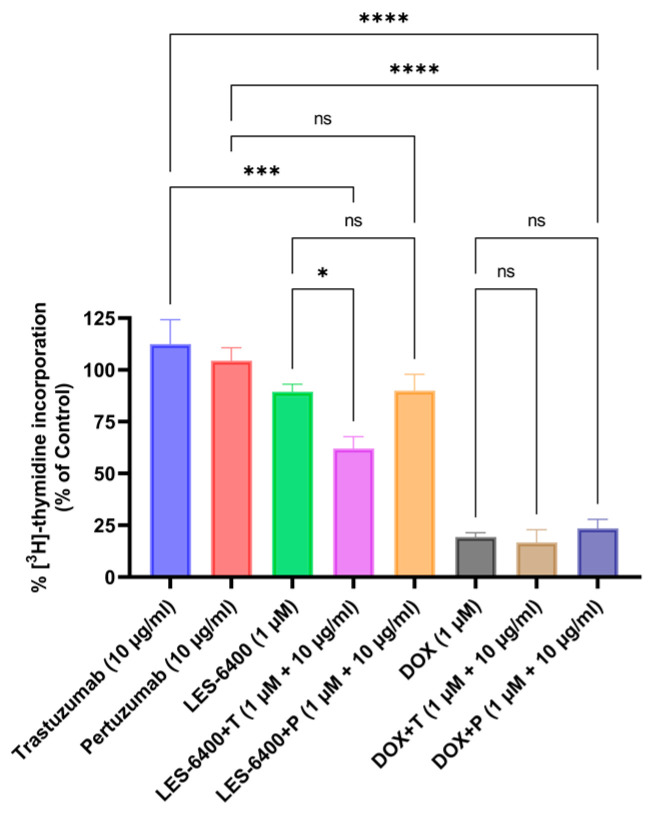
Incorporation of [^3^H]-thymidine into AGS gastric cancer cells was measured after 24 h of treatment with LES-6400 alone, trastuzumab alone, pertuzumab alone, DOX alone, and combinations of LES-6400 + T, LES-6400 + P, DOX + T, and DOX + P. Means ± SD are shown with *N* = 3. ANOVA tests were used to demonstrate differences between cells treated with compounds alone and the combination of compounds and antibodies. **** *p* ≤ 0.0001; *** *p* ≤ 0.001; * *p* ≤ 0.05; ns—not statistically significant.

The antiproliferative activity of the compounds and combination treatments tested against the AGS cell line is illustrated in the graph below (Figure 4). The results were consistent with those obtained in the MTT cytotoxicity assay. The tested LES-6400 derivative (1 μM) resulted in 89.39% ± 3.64% [^3^H]-thymidine incorporation relative to the control, trastuzumab (10 μg/mL) showed 112.49% ± 11.78%, and pertuzumab (10 μg/mL) showed 104.43% ± 6.27%. The combination LES-6400 + T (1 μM + 10 μg/mL) reduced [^3^H]-thymidine incorporation to 62.06% ± 5.66%, while the combination LES-6400 + P (1 μM + 10 μg/mL) resulted in 89.97% ± 7.849%. No significant differences were observed between monotherapy and combination therapy with the reference agent doxorubicin. These findings indicate that trastuzumab at a concentration of 10 μg/mL enhances the antiproliferative effect of LES-6400. In contrast, this effect is not observed with pertuzumab, which is why further studies were conducted exclusively with trastuzumab.

### 2.4. Clonogenic Assay

**Figure 5:**
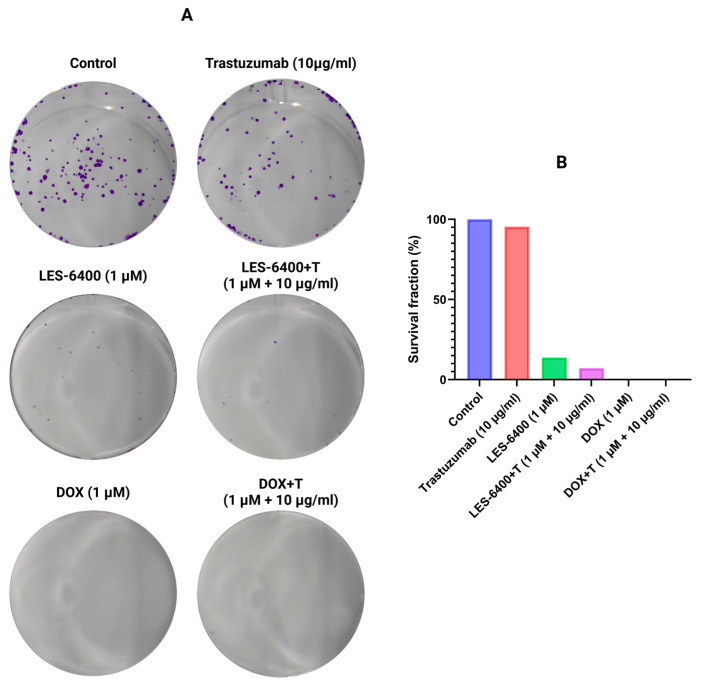
Representative cell culture wells (**A**) and graphical data (**B**) showing the results of the colony formation assay performed on AGS gastric cancer cells.

In addition to the MTT assay, the efficacies of treatment with the LES-6400 compound alone, trastuzumab alone, doxorubicin alone and the combinations LES-6400 + T and DOX + T were evaluated using the clonogenic assay. It was found that after 24 h of exposure, the survival fraction of cells treated with the combined therapy of LES-6400 (1 μM) and trastuzumab (10 μg/mL) (7.18%) was approximately 2 times lower than the fraction after exposure to LES-6400 (1 μM) alone (13.87%) and 13 times lower than after treatment with trastuzumab (10 μg/mL) alone (95.48%) (Figure 5). In contrast, the survival fraction of cells treated with doxorubicin, both with and without trastuzumab, was approximately 0%.

### 2.5. Trastuzumab Potentiates ROS Generation

**Figure 6:**
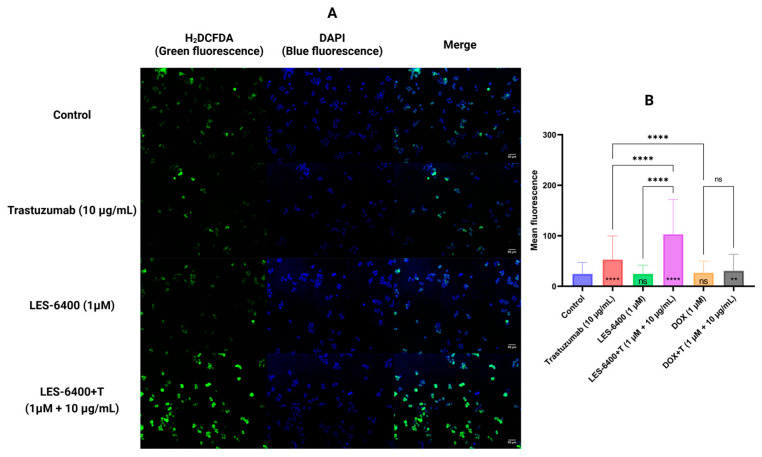
Results of H_2_DCFDA and DAPI double staining of AGS gastric cancer cells after 30 min of compound treatment. Representative fluorescence microscope images are shown (**A**). The graphs show the average fluorescence of each sample tested (**B**). ANOVA tests were used to show differences between control and compound treated cells (symbols in bars of graphs) and between cells treated with single compounds and their combinations with antibodies. *N* > 200; ** *p* ≤ 0.01; **** *p* ≤ 0.0001; ns—not statistically significant.

We performed double staining using H_2_DCFDA as an indicator of oxidative stress (green fluorescence) and DAPI to stain the nuclear genetic material of AGS gastric cells (blue fluorescence). This study allowed us to evaluate the generation of reactive oxygen species in the presence of the test compound, both as monotherapy and in combination therapy. Trastuzumab (10 μg/mL) in monotherapy caused an increase in ROS levels compared to the control after 30 min of treatment of gastric cells with AGS, which was not observed with the compound LES-6400 (1 μM) in monotherapy. In contrast, in the combination therapy LES-6400 + T, we observed an approximately twofold increase in ROS levels compared to trastuzumab monotherapy and a fourfold increase compared to control (Figure 6).

### 2.6. Trastuzumab Enhances the Proapoptotic Effect of the Compound LES-6400

**Figure 7:**
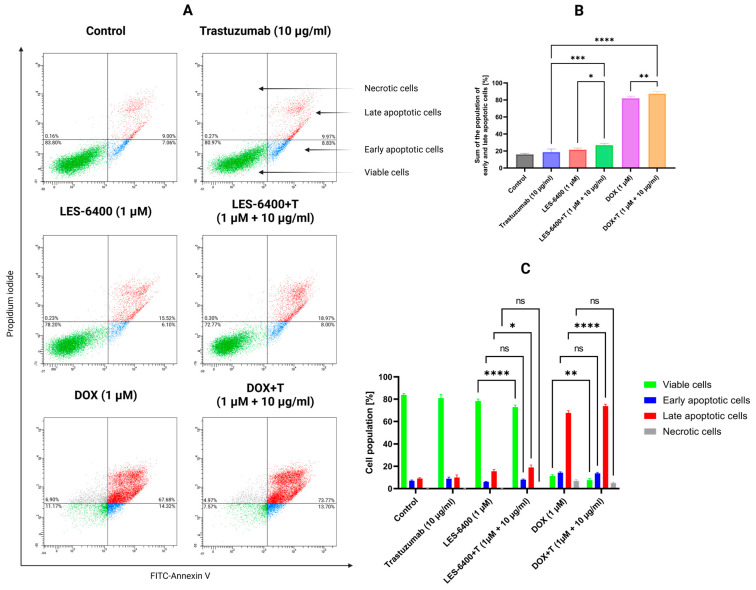
Results of flow cytometry analysis of AGS gastric cancer cells after 24 h of incubation with compound LES-6400 (1 μM), trastuzumab (10 μg/mL), DOX (1 μM), and the combinations LES-6400 + T (1 μM +10 μg/mL), and DOX + T (1 μM + 10 μg/mL) (**A**). The top graph (**B**) shows the totals of the apoptotic cell populations for each sample, while the bottom graph (**C**) visualizes the cytograms from the compound analysis. Means ± SD are shown with *N* = 3. ANOVA tests were used to demonstrate differences between cells treated with compounds alone and the combination of compounds and antibodies. * *p* ≤ 0.05; ** *p* ≤ 0.01; *** *p* ≤ 0.001; **** *p* ≤ 0.0001; ns—not statistically significant.

We used flow cytometry with the Annexin V-FITC and Propidium Iodide double staining (AV/PI) assay to qualitatively assess the process of programmed cell death, or apoptosis. This assay allows us to evaluate the population of cells treated with a given compound based on their state: unstained cells (alive), cells stained with propidium iodide (necrotic), cells stained with FITC-labeled annexin V (early apoptotic), and cells stained with both annexin V and propidium iodide (late apoptotic). For LES-6400, the AGS gastric cancer cell population consisted of 6.10% ± 0.42% early apoptotic, 15.53% ± 1.61% late apoptotic, and 0.23% ± 0.13% necrotic cells. For trastuzumab, these values were 8.83% ± 1.42% (early apoptotic), 9.97% ± 2.25% (late apoptotic), and 0.27% ± 0.21% (necrotic). With the combination LES-6400 + T treatment, we observed 8.00% ± 0.62% early apoptotic, 18.97% ± 2.14% late apoptotic, and 0.30% ± 0.00% necrotic cells. To better visualize our results, the graph below shows the sum of apoptotic cell populations for each sample (Figure 7). Our findings indicate the superiority of combination therapy over monotherapy in inducing programmed cell death in AGS gastric cancer cells (sum of apoptosis cells: LES-6400 + T – 26.97% vs. LES-6400—21.63%, trastuzumab—8.80%).

**Figure 8:**
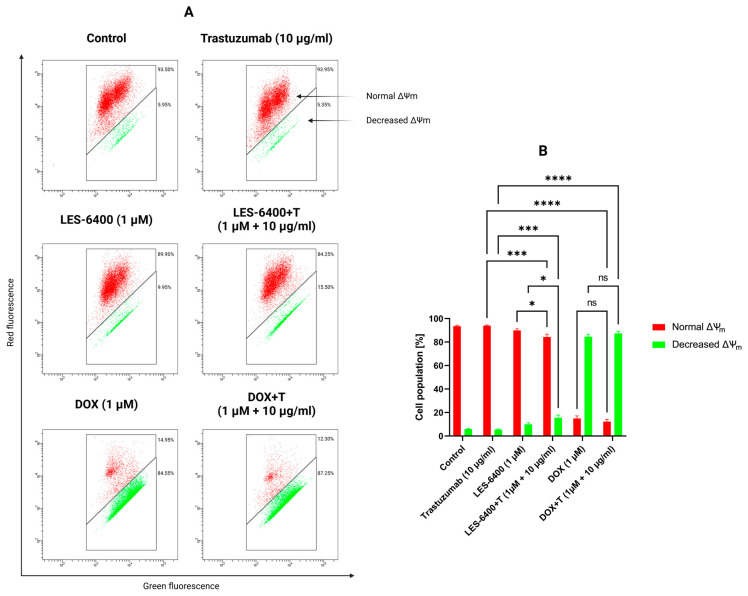
Results of flow cytometry measurement (**A**) of changes in the mitochondrial membrane potential (ΔΨm) of AGS gastric cancer cells after 24 h of incubation with compound LES-6400 (1 μM), trastuzumab (10 μg/mL), DOX (1 μM), and the combinations LES-6400 + T (1 μM + 10 μg/mL), and DOX + T (1 μM + 10 μg/mL). The graph (**B**) visualizes the cytograms from the compound analysis. Means ± SD are shown with *N* = 3. ANOVA tests were used to demonstrate differences between cells treated with compounds alone and the combination of compounds and antibodies. * *p* ≤ 0.05. *** *p* ≤ 0.001; **** *p* ≤ 0.0001; ns—not statistically significant.

Furthermore, the impact of monotherapy and combination therapy on mitochondrial membrane potential (ΔΨm) was examined. Abnormalities in mitochondrial membrane potential (MMP) may indicate induction of apoptosis by an intrinsic pathway. For this purpose, we used the dye JC-1, a fluorochrome that assumes different forms and emits different fluorescence depending on the MMP. In cells with normally functioning mitochondria, this dye forms aggregates that accumulate in the hyperpolarized mitochondrial membrane and emit red fluorescence. If the membrane is damaged, the dye breaks down into monomers, resulting in green fluorescence. The combination therapy LES-6400 + T (1 μM + 10 μg/mL) induced mitochondrial potential disruption in 15.50% ± 2.40% of the AGS gastric cancer cell population, whereas MMP disruption occurred in 9.85% ± 1.34% of the population with LES-6400 monotherapy (1 μM) and in 5.35% ± 0.50% of the population with trastuzumab monotherapy (10 μg/mL) (Figure 8).

**Figure 9:**
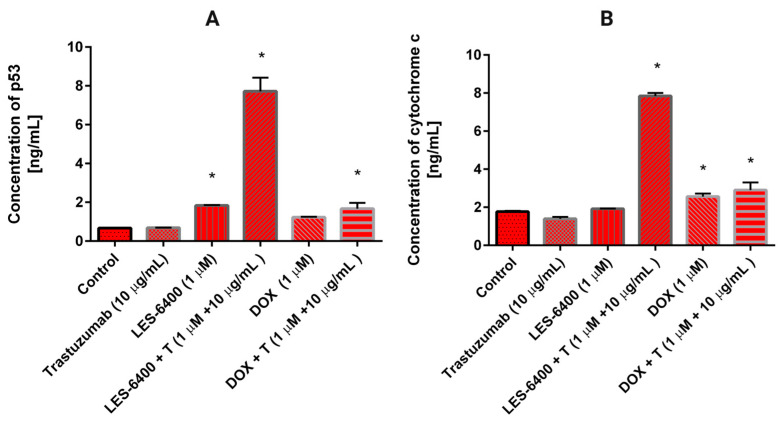
The concentration of p53 (**A**) and cytochrome C (**B**) in AGS gastric cancer cells after 24 h incubation with trastuzumab (10 μg/mL), LES-6400 (1 μM), LES-6400 + T (1 μM + 10 μg/mL), DOX (1 μM), DOX + T (1 μM + 10 μg/mL). The ANOVA and Tukey tests were used to demonstrate differences between the control cells and the cells exposed to varying concentrations of the tested compounds. Data presented in ng/mL. * *p* < 0.05 versus control group.

A statistically significant increase in p53 levels was observed in the presence of LES-6400, as well as in the combinations of LES-6400 + T and DOX + T (Figure 9A). p53 is a protein involved in regulating various cellular processes, particularly the activation of DNA repair mechanisms or the induction of apoptosis in response to DNA damage. In comparison to the control cells (0.67 ng/mL), the most notable elevation in p53 levels was observed with the combination of LES-6400 and trastuzumab (7.721 ng/mL). The elevation in p53 levels resulting from trastuzumab and doxorubicin treatments was comparable and not statistically significant. Conversely, LES-6400 exhibited a minimal yet significant impact on p53 levels in AGS gastric cancer cells, with an average value of 0.685 ng/mL. The distinction between monotherapy and combination therapy was notable, with combination therapy demonstrating enhanced efficacy.

The influence of the examined compounds on the level of cytochrome C, a protein implicated in the intrinsic apoptosis pathway that is released into the cytoplasm from the mitochondrial intermembrane space, was investigated in AGS gastric cancer cells. In the control cells that had not been subjected to any treatment, the concentration of cytochrome C was found to be 1.767 ng/mL (Figure 9B). The greatest increase in cytochrome C levels was observed following treatment with the combination LES-6400 + T, reaching 7.85 ng/mL. A 24-h incubation with DOX + T also elevated the cytochrome C concentration to 2.9 ng/mL, though this effect was less pronounced than that observed with the combination of the anti-HER2 antibody and LES-6400.

### 2.7. Determination of Inflammatory Cytokines

**Figure 10:**
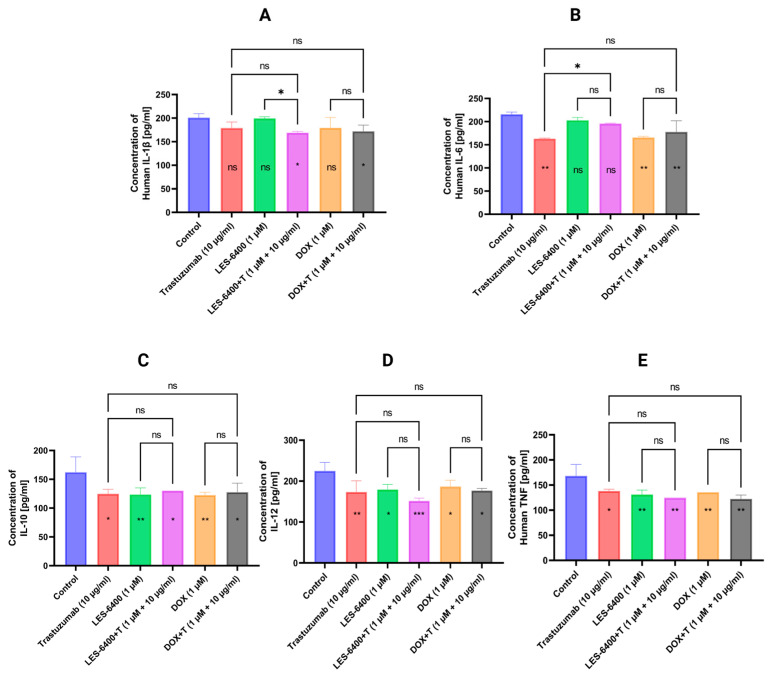
Graphs showing levels of inflammatory cytokine secretion (IL-1β (**A**), IL-6 (**B**), IL-10 (**C**), IL-12p70 (**D**), and TNF (**E**)) in AGS gastric cancer cells treated for 24 h with compound LES-6400 (1 μM), trastuzumab (10 μg/mL), DOX (1 μM), and their combinations. Graphs show mean ± SD with *N* = 3. ANOVA tests were used to show differences between control and compound treated cells (symbols in bars of graphs) and between cells treated with single compounds and their combinations with antibodies. * *p* ≤ 0.05; ** *p* ≤ 0.01; *** *p* ≤ 0.001; ns—not statistically significant.

To evaluate the inflammatory responses induced by compound LES-6400 (1 μM), DOX (1 μM), trastuzumab (10 μg/mL), and their combinations, we treated AGS gastric cancer cells with these compounds for 24 h and then evaluated the secretion of pro-inflammatory cytokines (IL-1β, IL-6, IL-10, IL-12p70, and TNF) by flow cytometry (Figure 10). Monotherapy with LES-6400 decreased the levels of TNF, IL-12p70, and IL-10, while IL-6 and IL-1β were not affected. In contrast, trastuzumab decreased the levels of all cytokines except IL-1β. Combination therapy with LES-6400 and trastuzumab caused a decrease in the levels of IL-1β, TNF, and IL-12, while the levels of IL-6 were not affected. The results of this study show that there is no statistical evidence of an advantage of LES-6400 combined with an antibody over LES-6400 or trastuzumab monotherapy. 

### 2.8. Combination of LES-6400 with Anti-HER2 Monoclonal Antibody Decreases the Concentration of IL-6 in Human Gastric Cancer Cells

The impact of trastuzumab (10 μg/mL), LES-6400 (1 μM), doxorubicin (1 μM), and their combinations (trastuzumab with LES-6400 or doxorubicin) on the concentration of IL-6 in AGS human gastric cancer cells was examined. To corroborate the findings obtained through flow cytometry, ELISA measurements were conducted.

**Figure 11:**
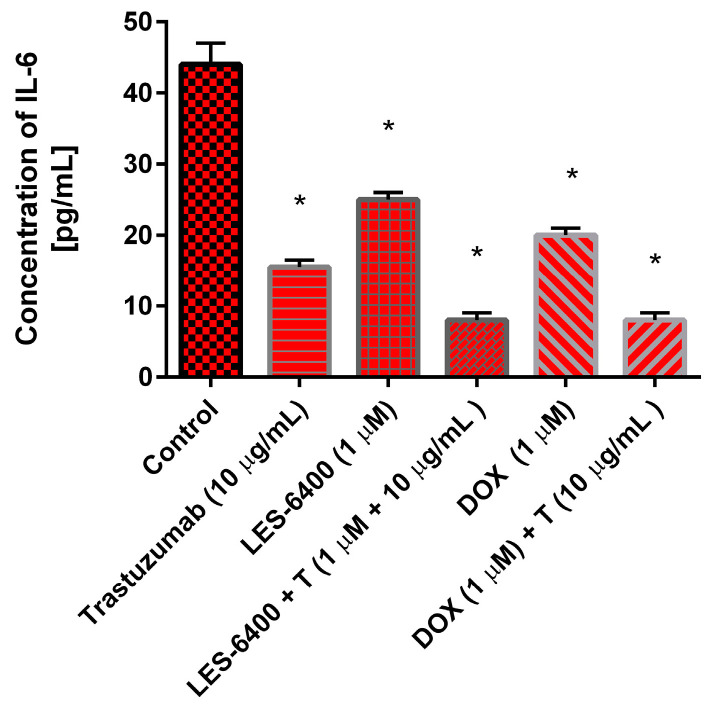
The concentration of interleukin-6 in AGS gastric cancer cells after 24 h incubation with trastuzumab (10 μg/mL), LES-6400 (1 μM), LES-6400 + T (1 μM + 10 μg/mL), DOX (1 μM), and DOX + T (1 μM + 10 μg/mL). The ANOVA and Tukey tests were used to demonstrate differences between the control cells and the cells exposed to varying concentrations of the tested compounds. Data presented in pg/mL. * *p* < 0.05 versus control group.

Both individual agents and their combinations with trastuzumab and chemotherapeutic agents demonstrated inhibitory activity against IL-6 (Figure 11). The administration of trastuzumab resulted in a reduction of the cytokine concentration from 44 pg/mL in the control cells to 15 pg/mL. At a concentration of 1 μM, LES-6400 reduced IL-6 levels to 25 pg/mL, while doxorubicin also resulted in an IL-6 concentration of 25 pg/mL. The combination strategy proved more effective in reducing pro-inflammatory cytokine levels, with the combination of trastuzumab with LES-6400 and doxorubicin significantly decreasing the IL-6 concentration to 8 pg/mL.

## 3. Discussion

In a previous study, our team assessed the efficacy of the combination of pertuzumab and trastuzumab with etoposide in the same AGS gastric cancer study model. The combination of etoposide with anti-HER2 antibodies (trastuzumab and pertuzumab) has been demonstrated to augment anti-proliferative effects and enhance apoptosis through the activation of intrinsic and extrinsic pathways, as evidenced by increases in caspase-8 and -9 levels and alterations in mitochondrial potential. The synergistic effects of both trastuzumab and pertuzumab in combination with etoposide were observed to be comparable, underscoring the potential therapeutic efficacy of this combination in the treatment of gastric cancer [27].

Our studies indicate that only trastuzumab at a concentration of 10 µg/mL causes a statistically significant enhancement of the effect of LES-6400 at a concentration of 1 µM against AGS gastric cancer cells. This was confirmed by cytotoxicity assays (trastuzumab: 97.95% ± 3.79%; LES-6400: 62.19% ± 0.44%; combination: 36.17% ± 11.27%) and by the assessment of [3H]-thymidine incorporation (trastuzumab: 112.49% ± 11.78%; LES-6400: 89.39% ± 3.64%; combination: 62.06% ± 5.66%). Pertuzumab did not enhance the anticancer activity. We did not observe significant changes in the HCC1954 breast cancer cell line. The efficacy of the combined therapy was also confirmed in a colony formation assay, where the survival fraction of cells in the combined therapy with trastuzumab was approximately two times lower than in the LES-6400 monotherapy and about thirteen times lower than in the trastuzumab monotherapy.

The findings suggest that the combination of the antiHER-2 antibody trastuzumab with LES-6400 enhances the intrinsic apoptotic pathway. Statistical analysis revealed a significant increase in apoptotic cell populations in the combination therapy group compared to the monotherapy group. Additionally, the combination therapy resulted in elevated levels of reactive oxygen species (ROS), altered mitochondrial membrane potential, and elevated levels of cytochrome C in the cytosol of cells, providing further evidence to support and confirm our observations.

Furthermore, a notable elevation in p53 protein levels was discerned in the context of combination therapy, in comparison to monotherapy. Cellular stress activates the tumor suppressor p53, which is a sequence-specific transcription factor that induces cell growth arrest or apoptosis. p53 stimulates a broad network of signals that act through both apoptotic pathways [28].

Interleukins, as immune mediators, are integral components of the immune system and are implicated in the pathogenesis of numerous diseases, including allergic reactions, autoimmune disorders, and cancer. Cancer cells can produce IL-1β, which is involved in tumor promotion and angiogenesis [29]. The results indicate that neither of the monotherapies showed statistically significant differences compared to the control group. In contrast, combination therapy with LES-6400 and trastuzumab resulted in a significant reduction in IL-1β levels.

Interleukin-6 (IL-6) has a multifaceted role in tumor promotion, including tumor progression, proliferation, migration, apoptosis, and angiogenesis. Elevated IL-6 levels are frequently associated with active disease states and poor prognosis [30]. ELISA assay demonstrated a significant reduction in IL-6 levels with trastuzumab monotherapy. However, flow cytometry did not show notable differences. In comparison, ELISA revealed that the decline in IL-6 levels with combination therapy of LES-6400 and trastuzumab was approximately five times less than with LES-6400 monotherapy and twice as much as with trastuzumab monotherapy.

The role of IL-10 in cancer remains controversial. Elevated IL-10 levels can stimulate tumor cell growth and proliferation, inhibit apoptosis, and facilitate immune evasion. Conversely, IL-10 can also exert tumor-suppressive effects by recruiting and stimulating cytotoxic T lymphocytes (CD8^+^ T) and natural killer (NK) cells, promoting immune memory, reducing the synthesis of pro-angiogenic factors, and decreasing the release of pro-inflammatory cytokines that contribute to tumor growth and invasion [31]. In our study, all treatment configurations resulted in a slight decrease in IL-10 levels.

IL-12 is a potent antitumor cytokine that enhances T-cell and NK-cell activity, reverses tumor-induced immunosuppression, inhibits angiogenesis, and induces interferon gamma (IFNγ) [32]. All combinations of the compound and antibody led to a reduction in IL-12 levels, with the combination of the compound and antibody causing a slight exacerbation of this decrease. 

Tumor necrosis factor (TNF) influences tumors by promoting metastasis and tumor growth [33]. In our study, all tested treatments led to a similar reduction in TNF levels.

Most of the parameters discussed above indicate that therapy with the juglone-based Thiopyrano[2,3-*d*]Thiazole derivative (LES-6400) in combination with an anti-HER2 antibody (trastuzumab) is more effective than therapy with these compounds alone.

## 4. Materials and Methods

### 4.1. Compounds

The anti-HER2 antibodies, trastuzumab and pertuzumab, were obtained from Selleckchem (Houston, TX, USA). The manufacturer’s declared purities were 99.7% and 99.17%, respectively. Doxorubicin hydrochloride (DOX) was obtained from Sigma-Aldrich (St. Louis, MO, USA).

The studied Thiopyrano[2,3-*d*]Thiazole derivative LES-6400 was designed, synthesized and identified according to protocol, based on our previous studies (in a previous article shown as compound 3.10) [13].

### 4.2. Cell Culture

Human gastric cancer (AGS CRL-1739™), human breast cancer (MCF-7 HTB-22™, MDA-MB-231 CRM-HTB-26™, HCC1954 CRL-2338™), and human colorectal adenocarcinoma (HT-29 HTB-38™, DLD-1 CCL-221™) cell lines were obtained from the American Type Culture Collection (ATCC, Manassas, VA, USA). The cell lines were cultured in complete medium: DMEM (Corning, Kennebunk, ME, USA)—AGS, MCF-7 and MDA-MB-231, RPMI-1640 (ATCC, Manassas, VA, USA)—HCC1954 and DLD-1, McCoy’s 5A (PAN-Biotech, Aidenbach, Germany)—HT-29. The media were supplemented with 10% fetal bovine serum (FBS) (Eurx, Gdansk, Poland) and 1% penicillin and streptomycin solution (Corning, Kennebunk, ME, USA). Cells were cultured in tissue culture dishes and cell culture flasks (Sarstedt, Numbrecht, Germany) at 37 °C, in an atmosphere containing 5% CO_2_, until they reached 90–95% subconfluence. For experiments, cells were then washed with phosphate buffered saline without calcium and magnesium (Corning, Kennebunk, ME, USA) and treated with 0.05% trypsin with 0.02% EDTA (Corning, Kennebunk, ME, USA). Cells were counted using a Scepter 3.0 cell counter (Merck Millipore, Burlington, MA, USA) prior to seeding into plates. The 6-well cell culture plates used 5 × 10^5^ cells/well in 2 mL of growth medium, while the 96-well plates (all plates from Sarstedt, Numbrecht, Germany) used 1 × 10^4^ cells/well in 100 μL of medium. Cells that reached approximately 80% confluence were used for further analysis.

### 4.3. Cell Viability Assay

The human cell lines were seeded into 96-well plates and cultured in accordance with the previously described methodology. Subsequently, the cells were treated for 24 h with LES-6400 or DOX at concentrations of 0.1, 1.0, 2.5, 5.0, 10.0, 25.0, and 50.0 μM, as well as their combinations with trastuzumab or pertuzumab (at 10 μg/mL). Following the selection of cell lines for further study, AGS and HCC1954 were tested in combinations of LES-6400, DOX, trastuzumab, pertuzumab, LES-6400 + trastuzumab, LES-6400 + pertuzumab, DOX + trastuzumab, DOX + pertuzumab. The MTT assay was conducted using a solution of 3-(4,5-dimethyl-2-thiazolyl)-2,5-diphenyl-2*H*-tetrazolium bromide (Sigma-Aldrich, St. Louis, MO, USA) at a concentration of 5 mg/mL in phosphate-buffered saline without calcium and magnesium (Corning, Kennebunk, ME, USA). Following a 24-h incubation period with the test compounds, 10 μL of the prepared MTT solution was added, and the cells were incubated for a further two hours at 37 °C. Subsequently, the cells were lysed in 100 μL of lysing buffer (comprising 25 mM HCl, 2% acetic acid, 3% DMF, and 5% SDS, with a pH of 4.7). The absorbance was determined at a wavelength of 570 nm using an Absorbance 96 microplate reader (Byonoy GmbH, Hamburg, Germany).

### 4.4. Evaluation of the Synergistic Effect of LES-6400 and Trastuzumab on AGS Gastric Cancer Cells

The combined effect of LES-6400 and trastuzumab on AGS gastric cancer cells was evaluated using the online tool SynergyFinder+ [34]. The tool employs four reference models: the Highest Single Agent (HSA) model, the Loewe Additivity Model, the Bliss Model, and the Zero Interaction Potency (ZIP) model. The HSA model postulates that the effect of a combination of drugs is equivalent to the superior effect of any of the drugs used individually. Synergy is defined as a combination that produces a greater response than any of the drugs used alone [35]. The Loewe model defines the expected effect of drugs as an additive response, assuming that the substances act as the same drug [36]. The Bliss model is based on the assumption that two drugs act independently in a stochastic process, whereby their effects can be analyzed as independent events. The expected effect of a drug combination is then calculated from the probabilities of these independent events [37]. The ZIP model is a method of calculating the effect of a combination of two drugs, assuming that neither drug affects the efficacy of the other. In this model, the drugs are treated as acting independently, allowing their interactions to be assessed in a way that does not consider each other’s effects on their effectiveness [38].

### 4.5. [^3^H]-Thymidine Incorporation Assay

Effects of LES-6400 (1 µM) and doxorubicin (1 µM), trastuzumab, and pertuzumab (10 µg/mL) and their combination on cell proliferation were evaluated. AGS cells were seeded in 6-well plates and cultured as described above. Cells were treated with the compounds and their antibody combinations and 0.5 μCi [^3^H]-thymidine was added for 24 h at 37 °C. The cells were then washed several times in PBS and precipitated by adding 0.5 mL/well of 5% trichloroacetic acid at 4 °C. After precipitation, the pellet was washed twice with 0.5 mL/well of 95% ethanol at 4 °C and then dissolved in 0.5 mL/well of 0.2 M NaOH. The dissolved precipitate was mixed with the scintillation solution and cell-associated radioactivity was measured using a Scintillation Counter 1900 TR, TRI-CARB (Packard, Perkin Elmer, Inc., San Jose, CA, USA). The [^3^H]-thymidine uptake was expressed as a percentage of the control value.

### 4.6. Clonogenic Assay

Cells were seeded in 12-well plates at 5 × 10^3^ cells/well in 1 mL medium. They were then left in an incubator for 24 h for attachment and adaptation. The next day, LES-6400 (1 µM), DOX (1 µM), trastuzumab (10 µg/mL), and their combinations with antibodies were added. After 24 h of treatment with the compounds, the medium was changed and the cells were allowed to form colonies for 7 days. After this time, the cells were fixed for 20 min at 4 °C and stained with crystal violet (0.01% in methanol). The wells were photographed and analyzed using ImageJ Version 1.50i software (Bethesda, MD, USA).

### 4.7. Reactive Oxygen Species Assessment

The effects of LES-6400 (1 µM), doxorubicin (1 µM), and trastuzumab (10 µg/mL) in monotherapy and combination therapy on the generation of reactive oxygen species (ROS) were evaluated. AGS cells were seeded in 6-well culture plates at 1 × 10^5^ cells per well in 1 mL medium. Cells were treated with compounds for 30 min and then washed with PBS. Double staining was performed with 20 µM 2′,7′-dichlorodihydrofluorescein diacetate (H2DCFDA) reagent (Thermo Fisher Scientific, Eugene, OR, USA), which is colorless, and is cleaved by intracellular esterases and oxidized to the highly fluorescent 2′,7′-dichlorofluorescein (DCF), and 10 µg/mL of the fluorescent dye 4′,6-diamidino-2-phenylindole (DAPI) (Sigma-Aldrich, St. Louis, MO, USA), which visualizes the nuclear DNA of cells. Observations were made with a Nikon ECLIPSE Ti fluorescence microscope (Nikon, Tokio, Japan). Results were analyzed using ImageJ software (Bethesda, MD, USA), where the fluorescence intensity of the DCF reagent was evaluated relative to the control. We tested >200 cells per sample. 

### 4.8. Influence on the Apoptosis

The effect of apoptosis induction by the tested compounds, antibodies and their combinations, used at the concentrations and incubation times described above, was assessed by staining with annexin V-FITC and propidium iodide (PI) using the Apoptosis Detection Kit II (BD Biosciences, San Diego, CA, USA). Cells were deprived of medium and washed several times with cold PBS. A cell suspension was prepared in the binding buffer provided in the kit at a concentration of 1 × 10^6^ cells/mL. From each sample, 100 µL of cell suspension was collected and transferred to tubes to which 5 µL of Annexin V-FITC and 5 µL of propidium iodide (PI) were added. The tubes were incubated for 15 min at room temperature in the dark. After incubation, the contents of the tubes were made up to 500 µL with a binding buffer and immediately read in a BD FACSCanto II flow cytometer (Becton Dickinson Biosciences, San Jose, CA, USA), where 10,000 events per sample were measured. Results were analyzed using FACSDiva Software Version 6.1.3. (BD Biosciences Systems, San Jose, CA, USA).

### 4.9. Effect on Mitochondrial Membrane Potential (MMP)

The lipophilic fluorochrome JC-1 (5,5′,6,6′-tetrachloro-1,1′,3,3′-tetraethylbenzimidazolcarbocyanine iodide) (BD Biosciences, San Diego, CA, USA) was used to assess the change in mitochondrial membrane potential. After treatment with the test compounds, antibodies and their combination, AGS gastric cancer cells were harvested at a concentration of 1 × 10^6^ cells/mL and washed twice with PBS. Then, 0.5 mL of the pre-prepared JC-1 working solution was added to each sample according to the manufacturer’s instructions. The samples were incubated for 15 min at 37 °C in a CO_2_ incubator. After incubation, samples were washed twice with an assay buffer and 0.5 mL of each buffer was transferred for analysis in a BD FACSCanto II flow cytometer (Becton Dickinson Biosciences, San Jose, CA, USA), where 10,000 events per sample were measured. Results were analyzed using FACSDiva software (BD Biosciences Systems, San Jose, CA, USA).

### 4.10. Determination of Inflammatory Cytokines

The evaluation of inflammatory cytokine concentrations (tumor necrosis factor [TNF], IL-1β, IL-6, IL-10, and IL-12) after exposure to the tested compound, antibody, doxorubicin, and their combinations was conducted using the Human Inflammatory Cytokines Kit (BD™ Cytometric Bead Array (CBA)) (BD Biosciences, San Diego, CA, USA). The assay was performed according to the manufacturer’s instructions on the collected supernatant from each sample. The samples were analyzed using a BD FACSCanto II flow cytometer (Becton Dickinson Biosciences, San Jose, CA, USA). The results were analyzed using FACSDiva software (BD Biosciences Systems, San Jose, CA, USA).

### 4.11. Determination of IL-6, p53, and Cytochrome C Concentration

Highly sensitive SimpleStep ELISA kits (Abcam, Cambridge, UK) were used to determine the levels of selected proteins in cell lysates after 24 h incubation with trastuzumab (10 μg/mL), LES-6400 (1 μM), doxorubicin (1 μM), and trastuzumab (10 μg/mL) in combination with LES-6400 (1 μM) or doxorubicin (1 μM). Briefly, after trypsinization, cells were washed three times with cold PBS and centrifuged at 1000× *g* for 5 min at 4 °C. Cells (1.5 × 10^6^) were then resuspended in a lysis buffer for whole cell lysates or supernatants. After the second centrifugation, the cell supernatants were immediately frozen at −70 °C. Untreated cancer cells were used as controls. The microtiter plates provided with the kits were precoated with an antibody specific for the antigen analyzed. Assays were performed according to the manufacturer’s protocols.

### 4.12. Statistical Analysis

GraphPad Prism Version 6.0 (San Diego, CA, USA) was used for all statistical analyses. Results are presented as mean ± standard deviation (SD). ANOVA and Tukey tests were used to show differences between samples exposed to different concentrations of the test compounds. Statistically significant difference was defined as * *p* ≤ 0.05; ** *p* ≤ 0.01; *** *p* ≤ 0.001; **** *p* ≤ 0.0001; ns—not statistically significant.

## 5. Conclusions

**Figure 12:**
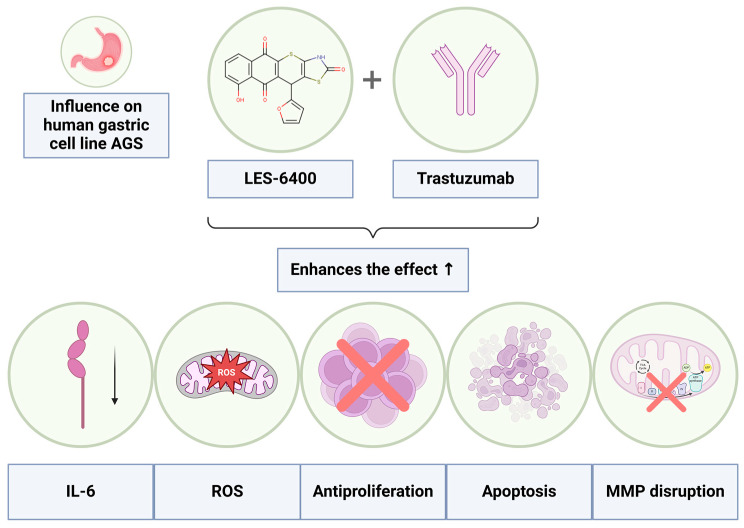
Graphical representation of the enhanced effects achieved through the combined therapy of the LES-6400 with the antibody trastuzumab.

The conducted studies demonstrated a significant advantage of the combined therapy using the juglone-based Thiopyrano[2,3-*d*]Thiazole derivative LES-6400 and the anti-HER2 antibody (trastuzumab) over monotherapies with these agents in the treatment of AGS gastric cancer. These results indicate a synergistic effect of both substances (Figure 12), leading to increased induction of apoptosis through disruption of mitochondrial membrane potential and potential induction of oxidative stress within cancer cells. Additionally, the combined therapy resulted in elevated levels of p53 protein and cytochrome C, which may suggest the activation of the intrinsic apoptotic pathway. Decreased IL-6 concentrations were also observed, indicating a modulation of the inflammatory response by the combined therapy.

The obtained results open new perspectives for optimizing combined therapies in gastric cancer treatment, particularly in the context of using novel heterocyclic compounds in conjunction with anti-HER2 monoclonal antibodies. However, further studies are needed to fully understand the mechanisms underlying the observed effects and to assess the potential clinical benefits of such a therapy.

## Figures and Tables

**Table 1:** IC_50_ ± SD values [µM] of tested compound and doxorubicin (DOX) as positive control after 24 h incubation with cancer cells.

Cell Line/Compound	LES-6400	DOX
AGS	1.88 ± 0.23	1.09 ± 0.23
DLD-1	2.10 ± 0.26	2.83 ± 0.47
HT-29	3.19 ± 0.41	0.73 ± 0.13
HCC1954	2.05 ± 0.38	3.17 ± 0.58
MCF-7	2.94 ± 0.62	0.55 ± 0.17
MDA-MB-231	10.02 ± 1.75	0.77 ± 0.23

## Data Availability

Data are contained within the article.

## Abbreviations

Akt	protein kinase B
Apaf-1	apoptosis protease-activating factor-1
CD8^+^ T	cytotoxic T lymphocytes
DAPI	4′,6-diamidino-2-phenylindole
DOX	doxorubicin
ELISA	enzyme-linked immunosorbent assay
FDA	The Food and Drug Administration
H2DCFDA	2′,7′-dichlorodihydrofluorescein diacetate
HER2	human epidermal growth factor receptor 2
IC50	half-maximal inhibitory concentration
IFNγ	interferon gamma
IL-10	interleukin-10
IL-12	interleukin-12
IL-1β	interleukin-1β
IL-6	interleukin 6
JC-1	5,5′,6,6′-Tetrachloro-1,1′,3,3′-tetraethylbenzimidazolylcarbocyanine, iodide
MMP	mitochondrial membrane potential
mTOR	mammalian target of rapamycin kinase
MTT	(3-(4,5-dimethylthiazolyl-2)-2,5-diphenyltetrazolium bromide) assay
NK	natural killer cells
p53	tumor protein P53
PARP1/2	poly ADP-ribose polymerase
PBS	phosphate buffered saline
PI3K	phosphoinositide 3-kinase
PPAR	peroxisome proliferator-activated receptors
ROS	reactive oxygen species
RTK	receptor tyrosine kinase
TNF	tumor necrosis factor
